# Expanding the Genomic Spectrum of *NHLRC2*-Associated FINCA Disease: Integrated Bioinformatic Characterization of a Novel Deep Intronic Variant Predicted to Activate a Pseudoexon

**DOI:** 10.3390/ijms27177555

**Published:** 2026-08-24

**Authors:** Anastasiia V. Rozhkova, Anton A. Esibov, Aleksandra N. Borkovskaia, Ekaterina A. Rutkovskaya, Olga S. Groznova, Olesya V. Sagaydak, Natalya A. Doroshchuk, Julia A. Krupinova, Olga N. Mityaeva, Mary Woroncow, Viktor P. Bogdanov, Pavel Y. Volchkov

**Affiliations:** 1 Federal Research Center for Innovator and Emerging Biomedical and Pharmaceutical Technologies, Ministry of Science and Higher Education of the Russian Federation, 125315 Moscow, Russiavolchkov_py@academpharm.ru (P.Y.V.); 2 Moscow Center for Advanced Studies, Ministry of Science and Higher Education of the Russian Federation, 123592 Moscow, Russia; 3 Veltischev Research and Clinical Institute for Pediatrics and Pediatric Surgery, Pirogov Russian National Research Medical University of the Ministry of Health of the Russian Federation, 125412 Moscow, Russia; 4 Charity Fund for Medical and Social Genetic Aid Projects «Life Genome», 119334 Moscow, Russia; 5 Department of Innovative Pediatrics and Pediatric Surgery, Pirogov Russian National Research Medical University of the Ministry of Health of the Russian Federation, 117997 Moscow, Russia; 6 National Medical Research Center of Cardiology Named After Academician E.I. Chazov, 121552 Moscow, Russia; 7 Evogen LLC, 115191 Moscow, Russia; 8 Moscow Clinical Scientific and Practical Center Named After A.S. Loginov, Moscow City Health Department, Novogireevskaya st., bldg. 1, 111123 Moscow, Russia; 9 Faculty of Medicine, Lomonosov Moscow State University, Leninskie Gory 1, 119991 Moscow, Russia

**Keywords:** *NHLRC2*, FINCA disease, deep intronic variant, pseudoexon inclusion, aberrant splicing, whole-genome sequencing, non-coding variant interpretation, genotype–phenotype correlation

## Abstract

*NHLRC2*-associated FINCA disease is an ultra-rare autosomal recessive multisystem disorder caused by biallelic pathogenic variants in *NHLRC2*. Its mutational spectrum and genotype–phenotype correlations remain incompletely defined, and the contribution of non-coding variants is poorly understood. Here, we report a male infant with a severe FINCA-like phenotype, including early-onset hemolytic anemia, pulmonary involvement, neurodevelopmental impairment, growth failure, recurrent infections, and fatal progression at 8.5 months. Whole-genome sequencing identified a compound heterozygous *NHLRC2* genotype comprising the previously reported pathogenic missense variant c.442G>T (p.Asp148Tyr) and a novel deep intronic variant, c.331+6863A>G. Segregation analysis confirmed inheritance from different parents. Integrated genomic and splicing analysis predicted that c.331+6863A>G creates a strong cryptic donor splice site and supports pseudoexon inclusion. Reconstruction of the predicted aberrant transcript indicated premature termination and potential susceptibility to nonsense-mediated mRNA decay. To our knowledge, this is the first reported deep intronic *NHLRC2* variant predicted to activate pseudoexon inclusion. Although experimental validation was unavailable, convergent clinical, segregation, population, and computational evidence supports c.331+6863A>G as the most plausible second disease-associated allele. This case expands the genomic spectrum of *NHLRC2*-associated FINCA disease and highlights the diagnostic value of phenotype-driven whole-genome sequencing.

## 1. Introduction

FINCA syndrome is an ultra-rare autosomal recessive disorder caused by biallelic pathogenic variants in the *NHLRC2* gene. The syndrome was first reported in 2018 by Johanna Uusimaa and colleagues in children presenting with severe pulmonary disease, neurological involvement, cerebral angiomatosis, and early death from respiratory failure [1]. Subsequent reports have expanded the clinical spectrum, demonstrating marked variability in age at onset, organ involvement, disease progression, and survival, including patients with substantially milder courses [2].

The *NHLRC2* gene encodes a protein thought to be involved in the regulation of oxidative stress [3] and phagocytic activity [4]; however, its precise biological function and the mechanisms underlying FINCA syndrome remain incompletely understood [2]. To date, only a limited number of affected individuals have been reported worldwide, and available data suggest considerable genotype–phenotype heterogeneity. Among the reported variants, c.442G>T (p.Asp148Tyr) represents a recurrent disease-associated allele. Notably, homozygosity for this variant has been described in patients with comparatively milder disease courses, including cases without prominent pulmonary involvement. These observations suggest that p.Asp148Tyr may retain partial functional activity and underscore the importance of documenting additional molecularly characterized cases to refine genotype–phenotype correlations.

Most reported *NHLRC2* disease-associated variants affect coding regions, whereas the contribution of non-coding variants to *NHLRC2*-associated FINCA disease remains unknown [2,5]. More broadly, deep intronic variants are increasingly recognized as a cause of Mendelian disease through aberrant splicing, including pseudoexon activation, and may require genome-wide approaches for detection and interpretation [6].

Here, we report a male infant with a severe early-onset multisystem phenotype consistent with *NHLRC2*-associated FINCA disease, who died at 8.5 months of age. Whole-genome sequencing identified a compound heterozygous *NHLRC2* genotype consisting of the recurrent pathogenic c.442G>T (p.Asp148Tyr) variant and a novel deep intronic variant, c.331+6863A>G. To our knowledge, deep intronic variants have not previously been reported as part of the molecular spectrum of *NHLRC2*-associated FINCA disease. This study aimed to evaluate the predicted molecular effect of this novel deep intronic *NHLRC2* variant using a computational framework combining phenotype-driven interpretation, whole-genome sequencing, and splicing analysis.

## 2. Results

### 2.1. Phenotype-Driven Clinical Evaluation

The proband was a male infant born at term by caesarean section due to signs of intrauterine hypoxia, including meconium-stained amniotic fluid. Apgar scores were 8 and 9 at 1 and 5 min, respectively. Birth weight was 2760 g (−1.08 SD), length was 48 cm (+0.74 SD), and head circumference was 33 cm (−0.62 SD). The neonatal period was complicated by indirect hyperbilirubinemia, leukocytosis, bilateral pneumonia, hemolytic anemia, and hypokalemia. The parents were non-consanguineous ethnic Tatars, and the family history was unremarkable, with one healthy older sibling.

From birth, the patient exhibited a severe multisystem phenotype dominated by hematologic, pulmonary, neurodevelopmental, gastrointestinal, and growth abnormalities. Hemolytic anemia was one of the leading manifestations. The anemia was normochromic and hyperregenerative, with a negative direct Coombs test, elevated lactate dehydrogenase, and reduced haptoglobin. No evidence of hematologic malignancy was found on bone marrow aspiration, which showed narrowing of the myeloid lineage and erythroid expansion. The patient experienced recurrent episodes of hemolysis during the first week of life, requiring repeated red blood cell transfusions.

Pulmonary involvement was clinically significant from early infancy. Interstitial lung disease was suspected during the first month of life, and differential diagnoses included Heiner syndrome and delayed transfusion-related acute lung injury. Serial chest computed tomography scans demonstrated diffuse ground-glass opacities with areas of consolidation, small cyst-like lesions, and compensatory emphysema in the upper lung fields. Bronchoscopy was unremarkable, while bronchoalveolar lavage showed increased mononuclear cells without siderophages. The patient had recurrent tachypnea, hypercapnia, and elevated lactate levels without persistent hypoxemia and remained oxygen-dependent during the first months of life.

Neurological and neuromuscular involvement included global developmental delay, hypotonia, dysphagia, and strabismus with a paralytic component developing at three months of age. Brain magnetic resonance imaging at 6.5 months revealed thinning of the corpus callosum. The patient also demonstrated progressive growth failure, with body weight reaching 5950 g (−3.41 SD) by 8 months of age. A trial elimination diet for suspected Heiner syndrome did not improve the leading clinical manifestations and was discontinued due to reduced weight gain. Abdominal ultrasound revealed moderate enlargement of the right hepatic lobe. Cardiac findings included two small secundum atrial septal defects and a patent ductus arteriosus with mild secondary pulmonary hypertension, without signs of right heart overload during follow-up.

The disease course was severe and progressive. The patient experienced recurrent infectious episodes, including two episodes of bacterial pneumonia and one episode of acute gastroenteritis; however, detailed immunological evaluation was not performed. At 8 months of age, bilateral multisegmental pneumonia and acute gastroenteritis were complicated by systemic inflammatory response syndrome and respiratory failure requiring mechanical ventilation. Despite antibacterial therapy, correction of electrolyte disturbances, vasopressor support, and intensive care treatment, the disease progressed to sepsis and multiple organ failure. The patient died at 8.5 months of age.

Taken together, the proband presented with an early-onset severe multisystem disorder characterized by Coombs-negative hemolytic anemia, interstitial lung disease-like pulmonary involvement, neurodevelopmental delay, hypotonia, growth failure, recurrent infections, and fatal progression during infancy. Alternative explanations considered during the diagnostic work-up, including Heiner syndrome, transfusion-related acute lung injury, hematologic malignancy, and metabolic disease, did not fully account for the persistent multisystem phenotype. Overall, the clinical presentation was consistent with a severe inherited multisystem disorder and prompted phenotype-driven genomic investigation. Following the identification of biallelic *NHLRC2* variants, the patient’s phenotype showed substantial overlap with the reported spectrum of *NHLRC2*-associated FINCA disease.

### 2.2. Molecular Findings

**Figure 1:**
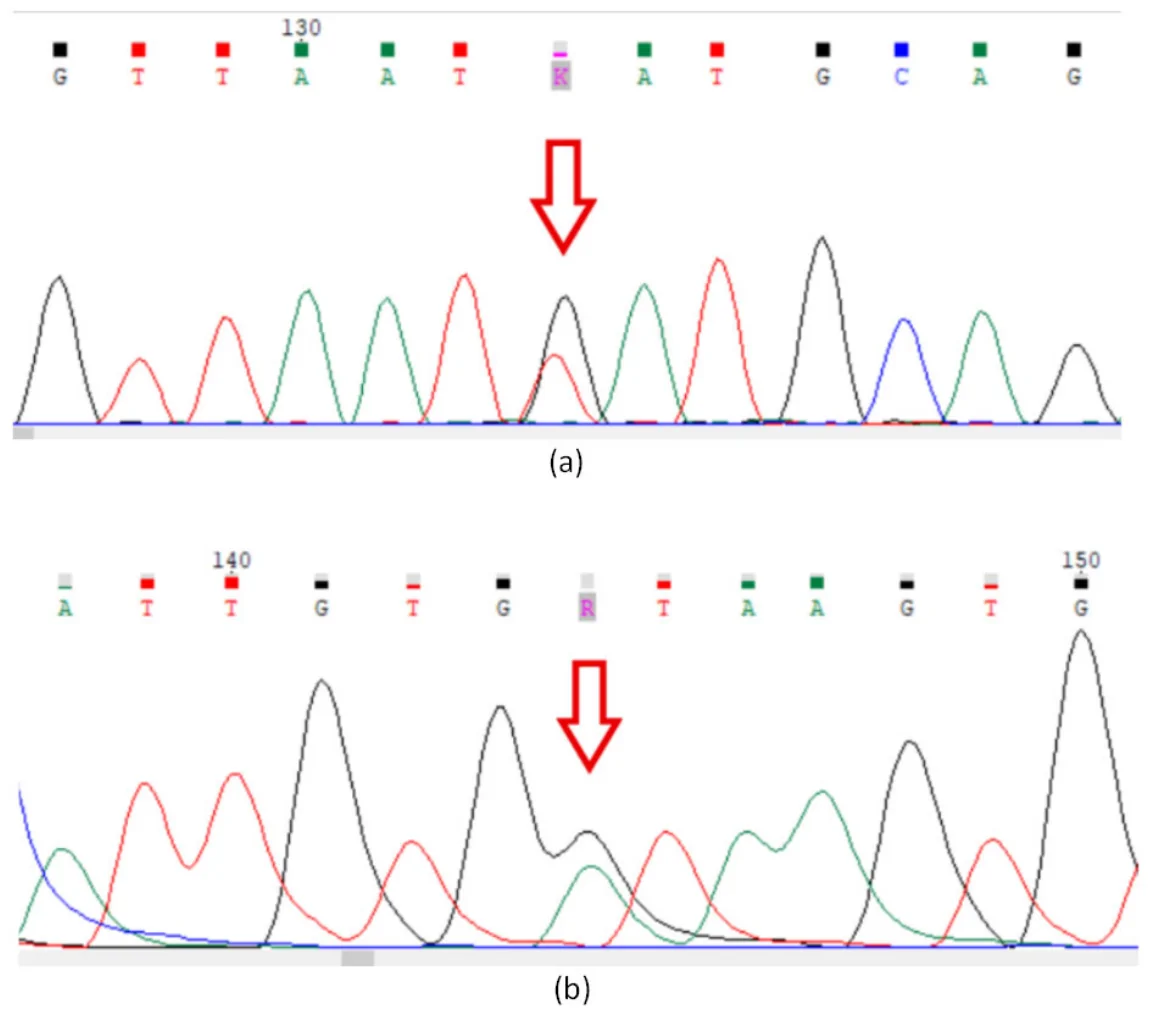
Segregation analysis of the *NHLRC2* variants by Sanger sequencing: (**a**) Electropherogram showing the heterozygous *NHLRC2* c.442G>T variant in the father. (**b**) Electropherogram showing the heterozygous *NHLRC2* c.331+6863A>G variant in the mother. The paternal and maternal origin of the two variants confirmed their compound heterozygous configuration in the affected proband.

Whole-genome sequencing identified two variants in the *NHLRC2* gene. Sanger sequencing confirmed both variants and clarified their parental origin. Segregation analysis by Sanger sequencing demonstrated that the variants were inherited from different parents: the father carried the coding variant *NHLRC2* (NM_198514.4):c.442G>T, whereas the mother carried the deep intronic variant *NHLRC2* (NM_198514.4):c.331+6863A>G. This segregation pattern established the compound heterozygous configuration of the variants (Figure 1).

The paternally inherited variant, *NHLRC2*(NM_198514.4):c.442G>T (p.Asp148Tyr), is located in exon 3. This variant has been previously described as pathogenic in association with *NHLRC2*-associated FINCA disease. Its allele frequency in gnomAD v4.1.0 is low, approximately 0.02%, with no homozygous individuals reported. In the Genome Russian Database (GDB v1.3.0), the variant was observed at a higher allele frequency of 0.11%, also without reported homozygous individuals. The pathogenicity of c.442G>T is supported by previous functional studies demonstrating significantly reduced *NHLRC2* protein expression. The variant is also present in ClinVar (Variation ID: 599377), where it was consistently classified as pathogenic by four submitters between 2021 and 2025. Based on the combined evidence, c.442G>T (p.Asp148Tyr) was classified as pathogenic according to ACMG/AMP criteria, meeting PS3_supporting, PM2_supporting, PM3_very strong, and PP3_supporting.

The maternally inherited variant, *NHLRC2*(NM_198514.4):c.331+6863A>G, is a deep intronic substitution located in intron 2 of *NHLRC2*. This variant has not been previously reported in patients with FINCA disease. It was absent from gnomAD v4.1.0 and was present in GDB v1.3.0 at a low allele frequency of 0.013%, with no homozygous individuals reported. At the initial stage of interpretation, the variant was considered a rare deep intronic variant of uncertain significance with potential relevance as a second *NHLRC2* allele because of its segregation in trans with a known pathogenic variant and the strong phenotypic overlap with *NHLRC2*-associated FINCA disease. No other convincing candidate variants consistent with the patient’s phenotype and the presumed inheritance model were identified in *NHLRC2* or in genes associated with overlapping clinical disorders.

Given the clinical concordance, autosomal recessive inheritance pattern, and segregation analysis confirming that the two *NHLRC2* variants were inherited in trans, the deep intronic c.331+6863A>G variant, in combination with the previously reported pathogenic c.442G>T variant, was prioritized as the most plausible molecular explanation of the patient’s disease.

Following further in silico splicing analysis and reconstruction of the predicted transcript consequence, the *NHLRC2* c.331+6863A>G variant was classified as likely pathogenic according to ACMG/AMP criteria, meeting PM2_supporting, PM3_moderate, PP3_supporting, and PP4_moderate.

### 2.3. Genomic and Splicing Analysis of the Deep Intronic NHLRC2 Variant

The novel *NHLRC2* variant c.331+6863A>G was located within intron 2 of the MANE Select transcript (NM_198514.4/ENST00000369301.3). Review of transcript annotations, including MANE Select/Plus Clinical, RefSeq Curated, RefSeq Predicted, and GENCODE Basic and Comprehensive tracks, showed no exon-level overlap between the affected intronic interval and annotated *NHLRC2* exons. Thus, the predicted 60-nucleotide inserted sequence was considered a candidate pseudoexonic sequence rather than a known alternative exon.

Analysis of the RepeatMasker annotation track in the UCSC Genome Browser [7] on the GRCh38 assembly did not reveal direct overlap between the predicted pseudoexon interval and annotated repetitive or transposable elements. The predicted pseudoexon was located at chr10:113,865,483–113,865,542. The nearest annotated repeats were outside this interval and included an upstream SINE element at chr10:113,864,948–113,865,248, a downstream simple repeat at chr10:113,865,561–113,865,583, and a downstream LINE element at chr10:113,865,598–113,865,662. These findings suggest that the predicted pseudoexon is unlikely to result from repeat-derived exonization and are more consistent with variant-driven activation of a cryptic donor splice site.

Conservation analysis did not reveal strong evolutionary constraint across the predicted pseudoexon. The variant position itself was not conserved (PhyloP = −0.106), whereas the predicted acceptor dinucleotide showed moderate local conservation (PhyloP approximately 1.34–1.40). Together with the local sequence context, these findings suggested that the region may contain a pre-existing cryptic acceptor site but is not part of a strongly conserved intronic element.

**Figure 2:**
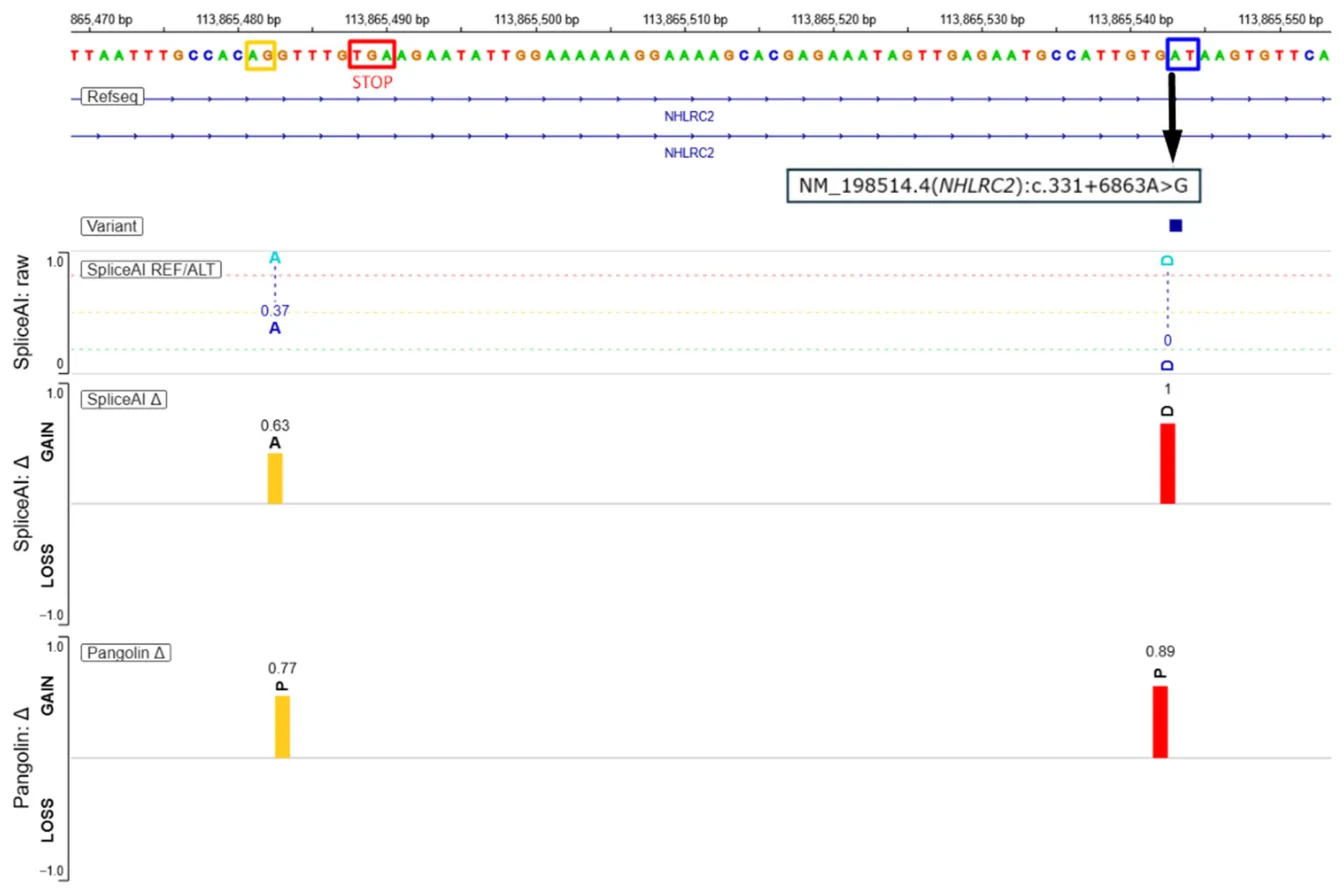
Image generated using the SpliceAI-lookup visualization tool. SpliceAI predicted activation of cryptic splice sites, including an acceptor gain (yellow filled rectangle) with a delta score of 0.63 and a donor gain (red filled rectangle) with a delta score of 1.0. Pangolin predictions supported the same splice-site architecture. The predicted arrangement of these sites is consistent with potential pseudoexon inclusion (60 bp) within the intronic region. The putative pseudoexon contains a premature termination codon, suggesting that the aberrant transcript would likely undergo nonsense-mediated mRNA decay. On the upper side of the image, the orange open rectangle corresponds to an active acceptor splice site, the red open rectangle corresponds to the potential stop codon, and the blue open rectangle denotes an active donor splice site.

Splicing prediction tools were used to assess the potential functional impact of the candidate deep intronic variant. SpliceAI and Pangolin concordantly predicted aberrant splice-site activation within the intronic region carrying c.331+6863A>G. The strongest signal corresponded to donor gain located 1 bp upstream of the variant position (GRCh38: chr10:113,865,542), with a SpliceAI [8] donor gain score of 1.00 and a Pangolin [9] donor gain score of 0.89, consistent with the creation of a strong cryptic donor site. Both tools also supported activation of an upstream cryptic acceptor site located approximately 60 bp upstream of the variant (GRCh38: chr10:113,865,483), with a SpliceAI acceptor gain score of 0.63 and a Pangolin acceptor gain score of 0.77. This acceptor–donor arrangement defined the predicted boundaries of an approximately 60-bp pseudoexon within intron 2 of *NHLRC2*. The predicted pseudoexon was flanked by a canonical upstream AG acceptor motif and a mutant downstream GT donor motif (Figure 2).

Reconstruction of the predicted aberrant transcript indicated that pseudoexon inclusion would introduce a premature TGA termination codon (Figure 2). Although the predicted pseudoexon is 60 nucleotides in length, its insertion occurs after coding position c.331, where the upstream coding exon ends with an incomplete codon. As a result, the inclusion of the pseudoexon is predicted to disrupt the coding sequence and generate a premature termination codon beginning at c.331+6808. The resulting transcript would be predicted to be susceptible to nonsense-mediated mRNA decay; alternatively, if translated, it would encode a severely truncated *NHLRC2* protein comprising 112 amino acids, compared with 726 amino acids in the full-length protein [10].

MaxEntScan [11] donor-site sliding-window analysis further supported this mechanism: at the predicted donor boundary, the c.331+6863A>G substitution increased the 5′ splice-site score from approximately 2 in the reference sequence to 10 in the mutant sequence (delta was +8.2), consistent with generation of a strong cryptic donor site. Together, these results support a model in which the wild-type sequence contains a cryptic upstream acceptor but lacks an efficient downstream donor site, whereas the c.331+6863A>G variant creates a strong donor motif and completes a pseudoexon-compatible acceptor–donor pair.

**Figure 3:**
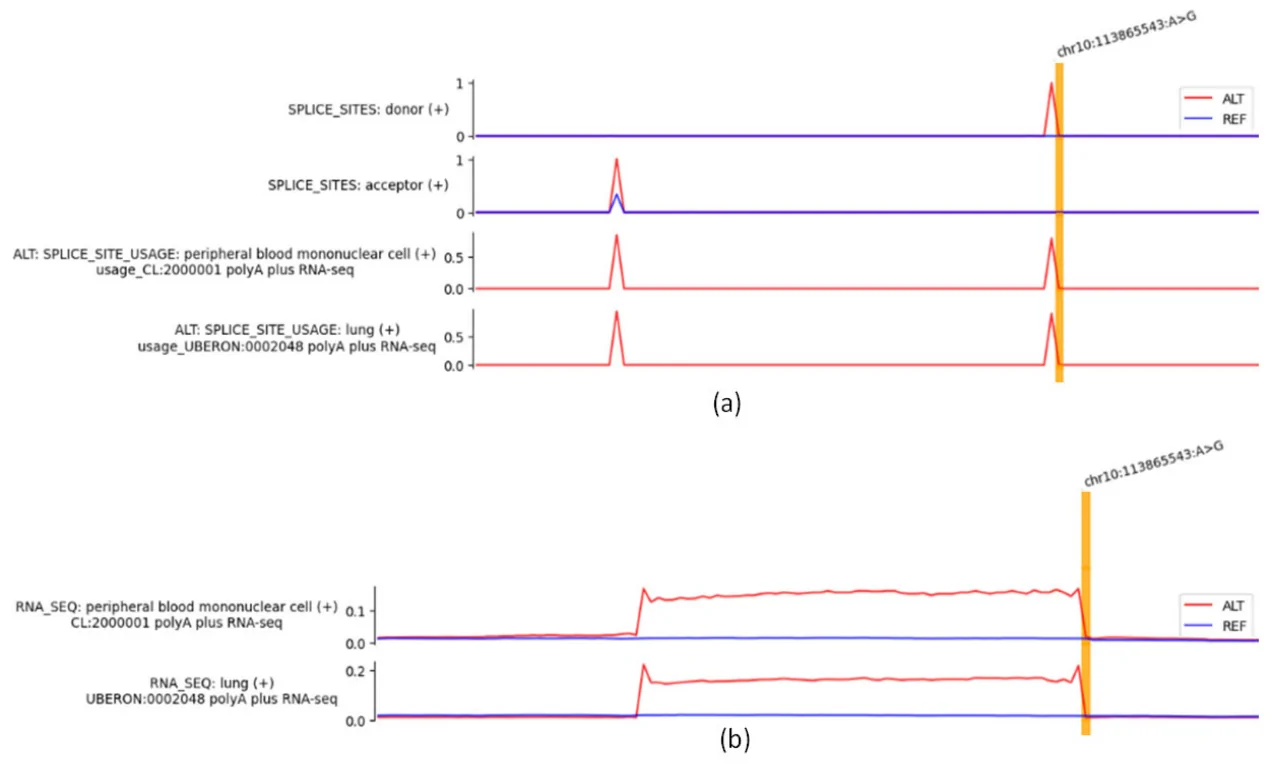
The genomic context of the *NHLRC2* intronic variant and AlphaGenome predictions for the reference and alternative alleles: (**a**) The alternative allele is predicted to increase cryptic splice site signals, including a putative acceptor and donor pair. Splice site usage predictions in lung and PBMC further support the potential use of these cryptic sites. (**b**) AlphaGenome-predicted RNA-seq coverage tracks are shown for lung and PBMCs. For both tissues, the reference allele shows minimal predicted coverage across the intronic region, consistent with normal intron removal, whereas the alternative allele shows increased predicted coverage over the putative pseudoexon (60-nucleotide sequence), supporting potential variant-associated pseudoexon inclusion. The vertical orange line corresponds to the nucleotide substitution c.331+6863A>G (localization of the new splice donor site).

AlphaGenome [12] predictions provided an additional, independent line of support for the proposed splicing effect (Figure 3). Compared with the reference allele, the alternative allele was predicted to increase cryptic splice-site signals corresponding to the putative acceptor–donor pair. Predicted splice-site usage and RNA-seq coverage tracks in lung and peripheral blood mononuclear cells showed minimal predicted intronic coverage for the reference allele, whereas the alternative allele showed increased predicted coverage over the 60-nucleotide pseudoexon. These findings were consistent with variant-associated pseudoexon inclusion.

**Figure 4:**
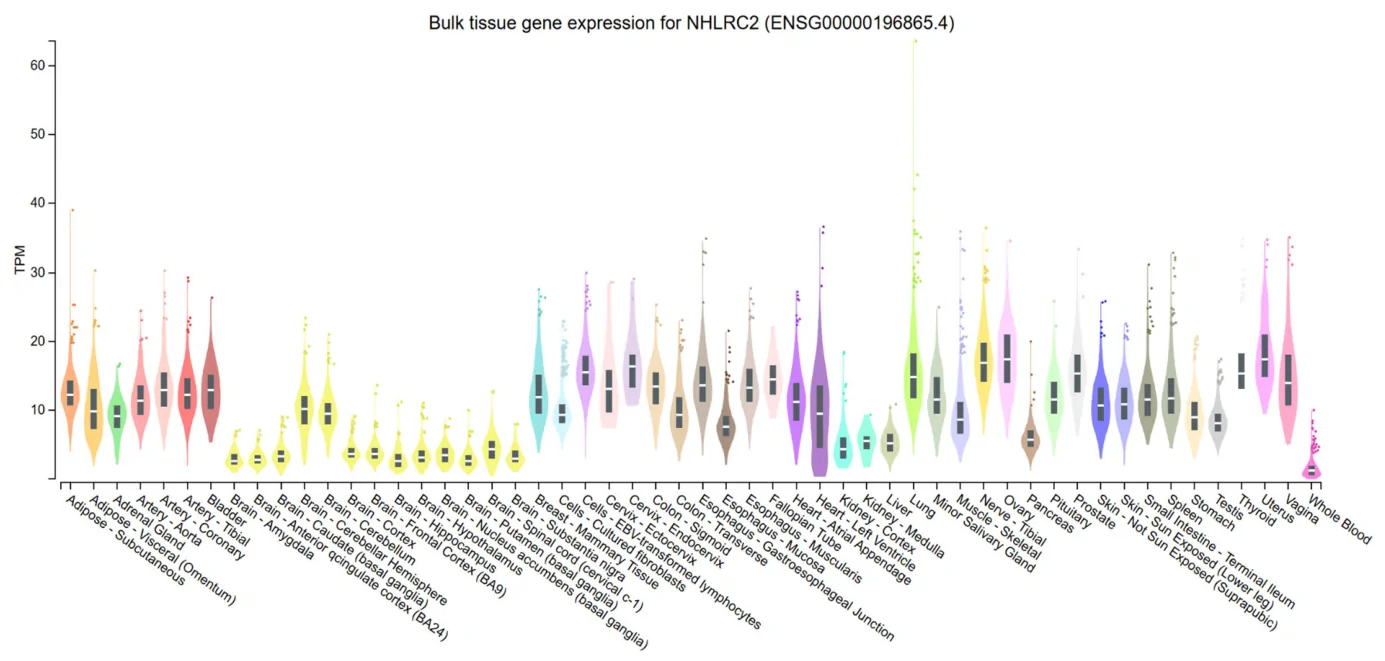
Tissue expression profile of *NHLRC2* according to GTEx data. *NHLRC2* showed broad tissue expression, with relatively high expression in lung and lower expression in brain compared with several other tissues. Expression in whole blood was among the lowest across tissues. This expression pattern is consistent with the multisystem nature of *NHLRC2*-associated FINCA disease and supports the tissue relevance of lung-based splicing predictions, while suggesting that peripheral blood RNA may be suboptimal for transcript-level validation. Expression values are shown as TPM (transcripts per million).

GTEx [13] tissue expression data were reviewed to contextualize the tissue relevance of the predicted splicing effect. *NHLRC2* showed broad tissue expression (Figure 4), with relatively high expression in lung and lower expression in brain compared with several other tissues. Expression in whole blood was among the lowest across tissues. This expression pattern is consistent with the multisystem phenotype and supports the tissue relevance of lung-based splicing predictions, while also suggesting that blood-derived RNA may be suboptimal for transcript-level validation.

Overall, the concordant SpliceAI and Pangolin predictions, motif-level MaxEntScan analysis, AlphaGenome predictions, and reconstruction of the predicted aberrant transcript supported a model in which the *NHLRC2* c.331+6863A>G variant creates a strong cryptic donor site and establishes a splice-site configuration predicted to support pseudoexon inclusion with premature termination. Experimental validation was not available; therefore, pseudoexon inclusion, premature termination, and nonsense-mediated decay remain predicted consequences.

## 3. Discussion

This study reports a severe infantile presentation of *NHLRC2*-associated FINCA disease and identifies a compound heterozygous *NHLRC2* genotype consisting of the known pathogenic missense variant c.442G>T (p.Asp148Tyr) and a novel deep intronic variant, c.331+6863A>G. The latter was prioritized as the most plausible second disease-associated allele based on its rarity, segregation from a different parent, concordance with the autosomal recessive inheritance model, and predicted effect on splicing. Integrated splicing analysis supported a model of variant-induced pseudoexon inclusion leading to premature termination and possible nonsense-mediated mRNA decay. Although experimental validation was not available, the convergence of clinical, segregation, population, and computational evidence supports the pathogenic relevance of the identified deep intronic variant.

The present case may contribute to emerging genotype–phenotype correlations in *NHLRC2*-associated FINCA disease. Previous reports of patients homozygous for the recurrent c.442G>T (p.Asp148Tyr) variant suggest that this allele is associated with a comparatively milder phenotype and may retain partial function. By contrast, the c.331+6863A>G allele described here is predicted to have a severe transcript-level consequence through pseudoexon inclusion, premature termination, and possible nonsense-mediated decay. The combination of a missense allele and a predicted loss-of-function intronic allele may therefore explain the more severe presentation observed in the proband. Nevertheless, additional cases and functional validation are required before definitive conclusions regarding allele-specific severity can be drawn.

A key finding of this study is the identification of a deep intronic *NHLRC2* variant predicted to activate pseudoexon inclusion. The molecular spectrum of *NHLRC2*-associated FINCA disease reported to date has mainly included exonic and canonical splice-region variants, including missense substitutions, nonsense and frameshift changes, and translation-initiation variants [1,2,5,14,15,16]. To our knowledge, deep intronic splice-altering variants have not previously been described in this disease. The present case therefore expands the genomic spectrum of *NHLRC2*-associated FINCA disease and indicates that non-coding intronic alleles should be considered when a highly concordant autosomal recessive phenotype remains only partially explained by coding-region findings [17].

This case also illustrates the diagnostic value of phenotype-driven whole-genome sequencing in severe multisystem rare diseases. The candidate second *NHLRC2* allele was located deep within an intron and would likely have been missed by exome sequencing or targeted panels, which primarily assess coding regions and selected exon–intron boundaries [6]. WGS can therefore be particularly informative when the clinical presentation strongly suggests a Mendelian disorder but coding-region analysis does not provide a complete molecular diagnosis, especially in infants and children with complex multisystem phenotypes [6]. At the same time, the identification of a deep intronic candidate variant is only the first step. Interpretation remains challenging and requires integration of inheritance model, segregation, population frequency, genomic context, splicing predictions, and predicted transcript consequence [17,18]. Thus, the present case supports both broader use of WGS in unresolved autosomal recessive disease and the need for more standardized approaches to prioritizing and interpreting deep intronic variants in clinical genome analysis [18].

A precise understanding of the molecular consequences of rare non-coding variants has implications beyond variant classification. In autosomal recessive disorders, mechanistic reconstruction can improve genetic counseling by clarifying recurrence risk, enabling targeted familial testing, and supporting reproductive planning. It may also guide the choice of functional validation strategies and, in the longer term, help identify splice-altering mechanisms that could be considered for therapeutic modulation. In the present case, the predicted pseudoexon activation mechanism provides a more specific diagnostic interpretation than variant rarity alone and illustrates how mechanistic analysis of deep intronic variants may contribute to more informative clinical genome interpretation.

GTEx expression data provided useful biological context rather than direct evidence of pathogenicity. Broad *NHLRC2* expression is consistent with the multisystem nature of FINCA disease, and relatively high expression in lung tissue is notable given the prominent pulmonary involvement in the proband. At the same time, GTEx reflects bulk RNA-seq from predominantly adult tissues and cannot capture developmental or cell-type-specific expression patterns relevant to infantile disease. The low expression of *NHLRC2* in whole blood also suggests that peripheral blood RNA may be suboptimal for transcript-level validation.

Several limitations should be acknowledged. First, pseudoexon inclusion was not experimentally confirmed. RNA-level or other functional validation, such as RT-PCR from patient material, targeted RNA sequencing, long-read RNA sequencing, or minigene analysis, was not available. Although a minigene splicing assay would be the most direct experimental approach to test whether *NHLRC2* c.331+6863A>G creates the predicted cryptic donor site and drives pseudoexon inclusion, its establishment would require additional experimental work beyond the scope of this retrospective case-based genomic study. Therefore, pseudoexon inclusion, premature termination, and nonsense-mediated decay remain predicted consequences. Second, the predicted pseudoexon interval was not strongly conserved, indicating that evolutionary conservation did not provide independent support for pathogenicity. This does not contradict the proposed mechanism, because the predicted effect is primarily driven by the creation of a strong mutant donor splice site rather than the disruption of a conserved intronic regulatory element. Third, this is a single-patient observation, and additional cases would be required to determine whether this deep intronic allele is recurrent, population-specific, or associated with a particular phenotypic severity. Experimental confirmation of the predicted splicing defect remains the key next step and may require minigene assays, targeted RNA sequencing, or patient-derived cells that are more informative than peripheral blood.

## 4. Materials and Methods

### 4.1. Clinical Data and Ethical Approval

Clinical data were obtained from the patient’s medical records and reviewed to assess the phenotypic spectrum, disease course, differential diagnoses considered during the diagnostic work-up, and concordance with previously reported manifestations of *NHLRC2*-associated FINCA disease.

The study was conducted in accordance with the Declaration of Helsinki and approved by the Independent Interdisciplinary Ethics Committee on Ethical Review for Clinical Studies (protocol no. 14, 21 July 2023). Written informed consent for genetic testing and publication of de-identified clinical and molecular data was obtained from the patient’s parents.

### 4.2. Whole-Genome Sequencing, Segregation Analysis, and Variant Classification

Genomic DNA was extracted from peripheral blood of the proband. Whole-genome sequencing was performed for the proband using a PCR-free library preparation protocol and paired-end 150 bp sequencing on the DNBSEQ platform, with a reported mean genome-wide coverage of at least 30×. Raw reads were processed using fastp v0.23.1 [19] and aligned to the GRCh38 human reference genome [20] using minimap2 v2.17 [21]. Variant calling was performed after base recalibration using GATK HaplotypeCaller v4.2 in single-sample mode [22]. Variant annotation and interpretation were performed using the laboratory’s internal clinical bioinformatics pipeline and relevant population and clinical variant databases, including gnomAD v4.1.0 [23], the Genome Russian Database (GDB v1.3.0) [24], ClinVar [25], OMIM [26], and relevant gene–disease resources.

Variant prioritization was performed using a phenotype-driven strategy, taking into account population frequency, gene–disease association, inheritance model, and concordance with the patient’s clinical phenotype. Candidate disorders with different modes of inheritance were evaluated separately. For autosomal recessive conditions, the analysis included the search for biallelic candidate variants compatible with the clinical presentation.

Sanger sequencing was performed to confirm the identified *NHLRC2* variants and determine their parental origin. Segregation analysis included both parents and was used to establish whether the two variants were inherited from different parental alleles.

Variant classification was performed according to ACMG/AMP guidelines [27]. The following evidence categories were considered: population frequency, previous reports in affected individuals, available functional evidence, computational predictions, allelic configuration, segregation data, phenotype specificity, and consistency with the known mechanism of *NHLRC2*-associated FINCA disease. For the deep intronic variant, computational splicing predictions and reconstructed transcript consequences were evaluated as supporting evidence; functional evidence criteria were not applied because transcript-level or other experimental validation was not available.

### 4.3. Genomic Context and Splicing Prediction

The deep intronic *NHLRC2* variant was interpreted relative to the MANE Select transcript NM_198514.4. Genomic coordinates were reported according to the GRCh38 assembly. Available transcript annotations in the UCSC Genome Browser and RefSeq were reviewed to assess whether the affected intronic interval overlapped annotated exons or represented a candidate pseudoexonic sequence.

Overlap with repetitive or transposable elements was assessed using the RepeatMasker annotation track available through the UCSC Genome Browser [7] on the GRCh38 assembly. Evolutionary conservation across the predicted pseudoexon interval and splice-site regions was assessed using vertebrate conservation tracks, including PhyloP and PhastCons scores, available through the UCSC Genome Browser [7].

Splicing effects of *NHLRC2* c.331+6863A>G were evaluated using complementary in silico tools, including SpliceAI [8] and Pangolin [9]. Predicted acceptor and donor gain signals were used to define the putative pseudoexon boundaries. The predicted donor site was additionally assessed using MaxEntScan sliding-window analysis [11] to compare 5′ splice-site scores between the reference and alternative alleles.

The predicted aberrant transcript was reconstructed by incorporating the putative pseudoexon, defined by the predicted acceptor and donor splice sites, into the reference *NHLRC2* transcript NM_198514.4. The reconstructed sequence was evaluated to determine the predicted coding consequence, the presence of a premature termination codon, and potential susceptibility to nonsense-mediated mRNA decay. *NHLRC2* protein length and annotation were reviewed using the UniProt [10] entry for human *NHLRC2*.

AlphaGenome [12] predictions were used as an additional computational assessment of the reference and alternative alleles at the *NHLRC2* c.331+6863A>G locus [ref]. Predicted splice-site usage and RNA-seq coverage tracks were reviewed for tissues relevant to the patient’s phenotype and potential transcript validation, including lung and peripheral blood mononuclear cells.

*NHLRC2* tissue expression was reviewed using the GTEx Portal based on GTEx v8 expression data [13].

## 5. Conclusions

This case expands the genomic spectrum of *NHLRC2*-associated FINCA disease by identifying a novel deep intronic variant predicted to activate pseudoexon inclusion. It also illustrates the diagnostic value of phenotype-driven whole-genome sequencing in severe autosomal recessive multisystem disease, particularly when coding-region findings do not provide a complete molecular explanation. Although experimental confirmation is still required, the convergence of clinical, segregation, population, and computational evidence supports *NHLRC2* c.331+6863A>G as the most plausible second disease-associated allele in this patient. More broadly, this case highlights the need for structured approaches to prioritizing and interpreting deep intronic variants in clinical genome analysis.

## Data Availability

Original contributions presented in this study are included in the article. Further inquiries can be directed to the corresponding author.

## Abbreviations

The following abbreviations are used in this manuscript:

ACMG/AMP	American College of Medical Genetics and Genomics/Association
DNA	Deoxyribonucleic acid
FINCA	Fibrosis, neuro-degeneration, cerebral angiomatosis syndrome
GATK	Genome Analysis Toolkit
GDB	The Russian Genetic Diversity Database
gnomAD	Genome Aggregation Database
GRCh38	Genome Reference Consortium Human Build 38
GTEx	Genotype-Tissue Expression portal
MANE	Matched annotation from NCBI and EMBL-EBI
mRNA	Messenger ribonucleic acid
OMIM	Online Mendelian Inheritance in Man
PBMC	Peripheral blood mononuclear cell
PCR	Polymerase chain reaction
RNA-seq	Ribonucleic acid sequencing
RT-PCR	Reverse transcription polymerase chain reaction
TPM	Transcripts per million
WGS	Whole-genome sequencing

## References

[1] Uusimaa J., Kaarteenaho R., Paakkola T., Tuominen H., Karjalainen M.K., Nadaf J., Varilo T., Uusi-Mäkelä M., Suo-Palosaari M., Pietilä I. (2018). *NHLRC2* Variants Identified in Patients with Fibrosis, Neurodegeneration, and Cerebral Angiomatosis (FINCA): Characterisation of a Novel Cerebropulmonary Disease. Acta Neuropathol..

[2] Tallgren A., Kager L., O’Grady G., Tuominen H., Körkkö J., Kuismin O., Feucht M., Wilson C., Behunova J., England E. (2023). Novel Patients with *NHLRC2* Variants Expand the Phenotypic Spectrum of FINCA Disease. Front. Neurosci..

[3] Nishi K., Iwaihara Y., Tsunoda T., Doi K., Sakata T., Shirasawa S., Ishikura S. (2017). ROS-Induced Cleavage of *NHLRC2* by Caspase-8 Leads to Apoptotic Cell Death in the HCT116 Human Colon Cancer Cell Line. Cell Death Dis..

[4] Haney M.S., Bohlen C.J., Morgens D.W., Ousey J.A., Barkal A.A., Tsui C.K., Ego B.K., Levin R., Kamber R.A., Collins H. (2018). Identification of Phagocytosis Regulators Using Magnetic Genome-Wide CRISPR Screens. Nat. Genet..

[5] Sczakiel H.L., Zhao M., Wollert-Wulf B., Danyel M., Ehmke N., Stoltenburg C., Damseh N., Al-Ashhab M., Balci T.B., Osmond M. (2023). Broadening the Phenotypic and Molecular Spectrum of FINCA Syndrome: Biallelic *NHLRC2* Variants in 15 Novel Individuals. Eur. J. Hum. Genet..

[6] Souche E., Beltran S., Brosens E., Belmont J.W., Fossum M., Riess O., Gilissen C., Ardeshirdavani A., Houge G., Van Gijn M. (2022). Recommendations for Whole Genome Sequencing in Diagnostics for Rare Diseases. Eur. J. Hum. Genet..

[7] Kent W.J., Sugnet C.W., Furey T.S., Roskin K.M., Pringle T.H., Zahler A.M., Haussler D. (2002). The human genome browser at UCSC. Genome Res..

[8] Jaganathan K., Kyriazopoulou Panagiotopoulou S., McRae J.F., Darbandi S.F., Knowles D., Li Y.I., Kosmicki J.A., Arbelaez J., Cui W., Schwartz G.B. (2019). Predicting Splicing from Primary Sequence with Deep Learning. Cell.

[9] Zeng T., Li Y.I. (2022). Predicting RNA Splicing from DNA Sequence Using Pangolin. Genome Biol..

[10] Ahmad S., Jose da Costa Gonzales L., Bowler-Barnett E.H., Rice D.L., Kim M., Wijerathne S., Luciani A., Kandasaamy S., Luo J., Watkins X. (2025). The UniProt Website API: Facilitating Programmatic Access to Protein Knowledge. Nucleic Acids Res..

[11] Yeo G., Burge C.B. (2004). Maximum entropy modeling of short sequence motifs with applications to RNA splicing signals. J. Comput. Biol..

[12] Avsec Ž., Latysheva N., Cheng J., Novati G., Taylor K.R., Ward T., Bycroft C., Nicolaisen L., Arvaniti E., Pan J. (2026). Advancing regulatory variant effect prediction with AlphaGenome. Nature.

[13] Aguet F., Anand S., Ardlie K.G., Gabriel S., Getz G.A., Graubert A., Hadley K., Handsaker R.E., Huang K.H., The GTEx Consortium (2020). The GTEx Consortium Atlas of Genetic Regulatory Effects across Human Tissues. Science.

[14] Rapp C.K., Van Dijck I., Laugwitz L., Boon M., Briassoulis G., Ilia S., Kammer B., Reu S., Hornung S., Buchert R. (2021). Expanding the Phenotypic Spectrum of FINCA (Fibrosis, Neurodegeneration, and Cerebral Angiomatosis) Syndrome beyond Infancy. Clin. Genet..

[15] Brodsky N.N., Boyarchuk O., Kovalchuk T., Hariyan T., Rice A., Ji W., Khokha M., Lakhani S., Lucas C.L. (2020). Novel Compound Heterozygous Variants in NHLRC2 in a Patient with FINCA Syndrome. J. Hum. Genet..

[16] Badura-Stronka M., Śmigiel R., Rutkowska K., Szymańska K., Hirschfeld A.S., Monkiewicz M., Kosińska J., Wolańska E., Rydzanicz M., Latos-Bieleńska A. (2022). FINCA syndrome—Defining neurobehavioral phenotype in survivors into late childhood. Mol. Genet. Genom. Med..

[17] Petersen U.S.S., Doktor T.K., Andresen B.S. (2022). Pseudoexon Activation in Disease by Non-splice Site Deep Intronic Sequence Variation—Wild Type Pseudoexons Constitute High-risk Sites in the Human Genome. Hum. Mutat..

[18] Ellingford J.M., Ahn J.W., Bagnall R.D., Baralle D., Barton S., Campbell C., Downes K., Ellard S., Duff-Farrier C., FitzPatrick D.R. (2022). Recommendations for Clinical Interpretation of Variants Found in Non-Coding Regions of the Genome. Genome Med..

[19] Chen S., Zhou Y., Chen Y., Gu J. (2018). Fastp: An Ultra-Fast All-in-One FASTQ Preprocessor. Bioinformatics.

[20] Schneider V.A., Graves-Lindsay T., Howe K., Bouk N., Chen H.-C., Kitts P.A., Murphy T.D., Pruitt K.D., Thibaud-Nissen F., Albracht D. (2017). Evaluation of GRCh38 and de Novo Haploid Genome Assemblies Demonstrates the Enduring Quality of the Reference Assembly. Genome Res..

[21] Li H. (2018). Minimap2: Pairwise Alignment for Nucleotide Sequences. Bioinformatics.

[22] McKenna A., Hanna M., Banks E., Sivachenko A., Cibulskis K., Kernytsky A., Garimella K., Altshuler D., Gabriel S., Daly M. (2010). The Genome Analysis Toolkit: A MapReduce Framework for Analyzing next-Generation DNA Sequencing Data. Genome Res..

[23] Chen S., Francioli L.C., Goodrich J.K., Collins R.L., Kanai M., Wang Q., Alföldi J., Watts N.A., Vittal C., Gauthier L.D. (2024). A Genomic Mutational Constraint Map Using Variation in 76,156 Human Genomes. Nature.

[24] Database of Population Frequencies of Genetic Variants of the Population of the Russian Federation. Federal Medical-Biological Agency of Russia. https://gdbpop.nir.cspfmba.ru/.

[25] Landrum M.J., Lee J.M., Benson M., Brown G., Chao C., Chitipiralla S., Gu B., Hart J., Hoffman D., Hoover J. (2016). ClinVar: Public Archive of Interpretations of Clinically Relevant Variants. Nucleic Acids Res..

[26] Amberger J.S., Bocchini C.A., Schiettecatte F., Scott A.F., Hamosh A. (2015). OMIM.Org: Online Mendelian Inheritance in Man (OMIM®), an Online Catalog of Human Genes and Genetic Disorders. Nucleic Acids Res..

[27] Richards S., Aziz N., Bale S., Bick D., Das S., Gastier-Foster J., Grody W.W., Hegde M., Lyon E., Spector E. (2015). Standards and Guidelines for the Interpretation of Sequence Variants: A Joint Consensus Recommendation of the American College of Medical Genetics and Genomics and the Association for Molecular Pathology. Genet. Med..

